# Continuous Accelerometry-Based Tremor Detection During Daily Living

**DOI:** 10.3390/s26051459

**Published:** 2026-02-26

**Authors:** Luis Martinez, Orlando Martinez, Stephen L. Schmidt, Rocio Rodriguez Capilla, Hector Gardea, Arabo Gholian, Dennis A. Turner, Deborah Soonmee Won

**Affiliations:** 1 Department of Electrical and Computer Engineering, California State University, Los Angeles, Los Angeles, CA 90032, USAomarti62@calstatela.edu (O.M.); hgardea@calstatela.edu (H.G.); agholia2@calstatela.edu (A.G.); 2 Department of Biomedical Engineering, Duke University, Durham, NC 27708, USA; stephen.schmidt@duke.edu (S.L.S.); dennis.turner@duke.edu (D.A.T.); 3 Department of Neurosurgery, Duke University Medical Center, Durham, NC 27710, USA; rocio.rodriguez@duke.edu

**Keywords:** adaptive DBS, Parkinsonian tremor, tremor detection, wearable accelerometer, implanted accelerometer

## Abstract

**Highlights:**

We developed an automated algorithm which detects Parkinsonian tremor during daily living activities using a commonly available commercial wearable accelerometer.

**What are the main findings?**

- Detects tremors on a second scale, whereas the currently known industry standard detects tremors on a minute scale.
- Distinguishes between voluntary physical activity and tremor.
- The output was highly correlated with the DBS intensity, such that detected tremor decreased as DBS intensity increased.

**What are the implications of the main findings?**

- Results from pilot testing our algorithm demonstrate the feasibility of practically implementing continuous tremor detection for Parkinson’s patients with deep brain stimulation (DBS) using a commercially available, convenient, wrist-worn watch.
- Continuous tremor estimates on a seconds resolution will enable adaptation of brain stimulation based on the patient’s current tremor state.

**Abstract:**

As a step towards advancing adaptive DBS control for Parkinson’s disease, we have developed an automated algorithm that detects tremor continuously on a seconds-resolution time scale from a wearable accelerometer and present the feasibility study test results. Triaxial acceleration data were wirelessly streamed from an Apple Watch as well as acquired from an internal accelerometer in the implanted DBS device itself. The algorithm first determines if there is any high-power voluntary activity, such as walking, using the arm, or transitioning from sitting to standing; then, it identifies peaks in the 4–7 Hz Parkinsonian tremor frequency band. Peak detection for tremor activity was more accurate using the Apple Watch than the IPG. Peak and harmonic detection were also more accurate using continuous wavelet transforms than short-time Fourier transform. According to the repeated measures correlation, our detection algorithm correlated strongly with DBS intensity (Subject RZCH: r = −0.93, *p* = 3.6 × 10^−5^; 6KOZ: r = −0.97, *p* = 1.6 × 10^−5^, NU5U: r = −0.94, *p* = 0.057). Pearson’s correlation coefficient between our tremor detection algorithm and the currently accepted industry metric was found to be 0.57 (t-value = 8.5, dof = 148, *p* < 1 × 10^−6^). Our algorithm is distinctive in the capability to detect Parkinsonian tremor, with a high degree of clinical relevance, during daily living activities and is able to discriminate tremor from walking, using a convenient, commercial wrist-worn accelerometer.

## 1. Introduction

Tremor is a prominent symptom of Parkinson’s disease [1,2,3] and has been effectively treated by deep brain stimulation (DBS) [4,5,6,7], but not always reliably or without side effects [8,9,10]. Researchers are working toward developing adaptive DBS (or aDBS), DBS with closed-loop control to adapt the stimulation settings (e.g., frequency, intensity, and which contacts to use for stimulation) based on biomarkers according to the current patient state, such as resting, standing, or walking [11,12]. The ability to detect tremor (and patient state) continuously would advance DBS technology towards aDBS, which would in turn provide a more power efficient and effective DBS therapy [13]. To guide effective treatment of Parkinsonian symptoms, biomarkers need to be identified which serve as quantitative, objective metrics of clinical outcome and medical status; for Parkinson’s disease, these metrics entail measures of symptom severity. Current metrics for parameter adjustment are limited by subjectivity and the inability to practically monitor the symptoms continuously, as a trained physician is required to assess the symptoms at infrequent doctor’s visits [14,15]. The physician provides a tremor rating between 0 and 4 based on their observations of multiple types of tremor, in the standardly used Unified Parkinson’s Disease Rating Scale (UPDRS) [16]. There has been work to develop a more objective, quantitative metric of tremor, particularly using accelerometer-based measurements [15].

Thus far, aDBS is being explored to control stimulation amplitude, and most of these algorithms are based on the presence of neural oscillations [17,18]. The beta oscillation is believed to be a biomarker for PD symptoms of akinesia and rigidity [1,2,3]. No neural biomarker has yet been identified for tremor, although some studies implicate the possibility of using neural biomarkers for tremor [2,19]. A couple recent investigations have explored local field potentials to attempt aDBS to control tremor: one investigation was applied to essential tremor not PD, but more importantly, rather than detecting tremor, the algorithm detected “tremor-evoking movements” with a 20% missed detection rate; the main result was the ability to substantially reduce power consumption while stimulating during 80% of tremor-evoking movements [20]. Another was able to detect tremor from local field potential (LFP) spectral power with a goodness of fit of R^2^ = 0.429 and these detections were carried out on intraoperative LFPs [19]; detection performance is unknown for tremor occurring during daily living activities. Still others are developing methods to discriminate between different tremor disorders but these are not methods that would be feasibly implemented in real time during daily living activities [21,22,23].

Tremor has been detected in previous studies [24,25]. Three key aspects distinguish our study from prior studies. In this feasibility study, we (1) test a wearable accelerometer against an accelerometer embedded within the implantable pulse generator (IPG), or DBS device itself; (2) compare detections by our algorithm against the current industry standard in DBS related software, the Apple movement disorders kit (MDK) as implemented by Rune Labs [26], and demonstrate that we can detect tremor at a much higher temporal resolution than this current industry standard; and (3) demonstrate that our metric correlates with DBS intensity for various electrical “doses”.

## 2. Materials and Methods

### 2.1. Experimental Protocol

This study was performed as part of a clinical feasibility trial for which FDA approval was obtained under IDE # G180280 and registered with clinicaltrials.gov (#NCT03815656) [13]. The protocols used for this study were approved by the Medtronic External Research Program Board and Duke University Health System Institutional Review Board, in addition to the FDA. All participants provided written informed consent. Study participants (limited to *n* = 5 for the feasibility study) were implanted with bilateral deep brain stimulation leads in both the subthalamic nucleus (STN) and globus pallidus (GP) for treatment of Parkinson’s disease. Patient demographics are provided in Table 1. They were each implanted with Medtronic’s Summit RC+S device (Medtronic Inc., Minneapolis, MN, USA). In the Summit RC+S device, the implantable pulse generator (IPG), which is the implanted device housing all the electronics which control the stimulation itself for DBS therapy, contains a triaxial accelerometer. The IPG is implanted subcutaneously just below the clavicle. Each patient subject was also given an Apple Watch (Apple Inc., Cupertino, CA, USA) which also contains a triaxial accelerometer at the wrist. The participants wore the watch on their tremor-dominant side; the two who did not exhibit tremor wore the watch on their dominant-use side. All participants wore the watch on the same individualized side across the dataset presented here. The Apple Watch accelerometer data are wirelessly streamed to a cloud server customized by Rune Labs (San Francisco, CA, USA) for storing and analyzing data from the Apple Watch.

At their monthly research visits in the clinic, the participants were instructed to carry out a series of activities for approximately 30 s at a time while accelerometry data were acquired both from the Apple Watch as well as the IPG: (1) sit, (2) stand, (3) walk, (4) sit, (5) sit while texting; thus, each recording was approximately 2.5 min in duration. A log was electronically recorded with more precise timing of the transitions between activities; custom code was written in C# (Microsoft, Redmond, WA, USA) for the clinical investigator to encode the time at which the participant transitioned to the next 30 s epoch of activity and record that time stamp in a text file. The MATLAB (R2023a, Mathworks, Natick, MA, USA) code that was written to implement our tremor detection algorithm then was able to parse the text file for these time stamps. At least two trials were performed, including one at clinical amplitude (100%) and one trial at reduced amplitude (typically 60 or 80% of clinical amplitude). Reduced amplitudes were selected to allow the presence of some symptoms but not to the degree that would make symptom levels unsafe to perform the walking epoch in the participants’ estimation.

### 2.2. Tremor Detection Algorithm

**Figure 1:**
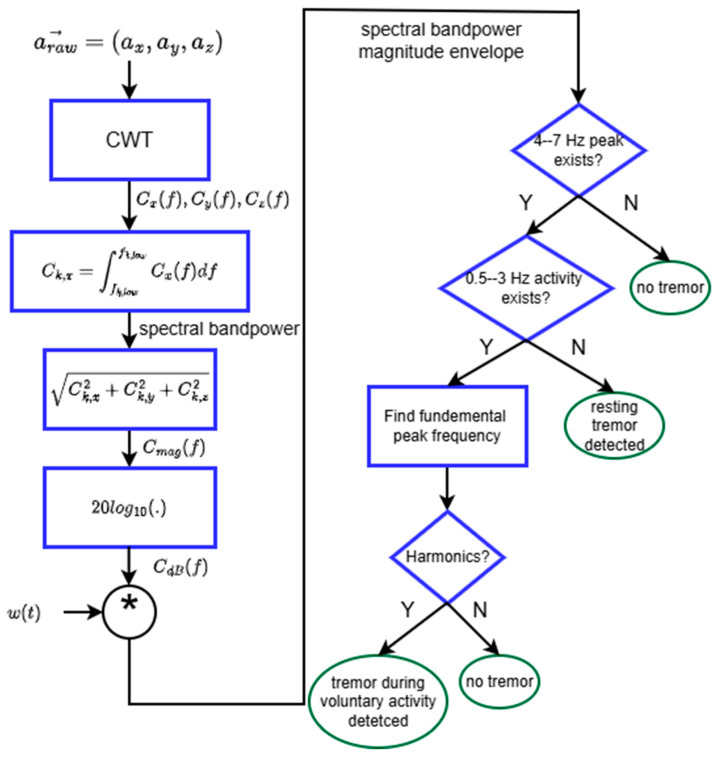
Flow chart of the tremor detection algorithm. The * symbolizes convolution.

The tremor detection algorithm is depicted by the flow chart in Figure 1. The tremor classification is based on spectral features. The main objective of our algorithm is to determine if there is a peak in the 4–7 Hz band of the acceleration signal’s spectrum and confirm whether the peak is due to tremor or is a harmonic of other physical activity, such as walking.

The spectral components Cx(f), Cy(f), and Cz(f) were obtained by applying a continuous wavelet transform to the raw acceleration ax, ay, and az. The spectrum was broken into physiologically relevant frequency bands (0.5–3 Hz and 4–7 Hz). Band power for the kth frequency band was found by trapezoidal numerical integration using MATLAB’s built-in trapz function. The magnitude of the spectral band power in dB scale was then convolved with a rectangular window of duration 1.5 s to obtain the moving average envelope of the spectrum. Thus, the overall latency expected in detecting tremor is 1.5 s. MATLAB’s islocalmax [27] was applied to the moving average to determine if there was a peak with a minimum prominence of an empirically determined threshold of 12 in the 4–7 Hz band; the other parameters for islocalmax were set to the default values. If not, the algorithm determined that no tremor was present. If there was 4–7 Hz activity, and there was also 0.5–3 Hz activity, we determined if the patient was walking by searching for the fundamental frequency of the 0.5–3 Hz activity peak. If there were harmonics of the fundamental peak in the 0.5–3 Hz band (i.e., peaks at integer multiples of the fundamental frequency were present), we would detect that the patient was carrying out voluntary physical activity, such as walking or transitioning between sitting and standing. We then determined if there were any other 4–7 Hz peaks, in which case the algorithm would detect that the patient had tremor while performing walking or transitioning between activities. If there was no 0.3–3 Hz peak, the algorithm would check for any 4–7 Hz peaks, in which case it would detect that the patient was experiencing tremor; if not, it would detect that there was no tremor and no higher energy activity (such as walking or transitioning between states).

The spectral components were obtained using two different methods and the quality of the spectrogram with the two methods was compared. The first was short-time Fourier transform (STFT) implemented with MATLAB’s built-in stft function, using a 1.5 s Hanning window and 70% overlap, which equates to updates in the spectral features and tremor detection output every 450 ms, with a 1.5 s delay. The second was the continuous wavelet transform which was implemented with MATLAB’s cwt function using the Morse wavelets with a symmetry parameter γ = 3 and time-bandwidth product P^2^ = 60.

Rune Labs created a platform called StrivePD for streaming data from the Apple Watch, cloud storage of the data, and viewing and processing the data. IPG triaxial accelerometry measurements were acquired at a sampling rate of 65.1 samples/s, while the Apple Watch acceleration was sampled at 50 samples/s, although packets were sometimes lost in transmission, but time stamps were recorded along with each sample. Other data acquisition parameters are proprietary. To synchronize the IPG and Apple accelerometry signals, we used custom MATLAB code to import each session’s IPG .json file and parse the aforementioned clinician log for the time stamp of the first sample at the start of each session. Then, we extracted the corresponding time segment from the Apple acceleration stream. Because the IPG accelerometry had a higher sampling rate overall and to preserve the temporal resolution, we created a master time vector to which we interpolated the Apple acceleration using MATLAB’s interp1 command. StrivePD utilizes the Apple movement disorders kit (MDK) to provide continuous read outs on the presence of tremor [28]. In the Apple MDK, tremor severity is quantified as the percentage of time over 1 min periods that the patient is exhibiting tremor. This is one of several tremor metrics for Parkinson’s disease patients implemented in the industry; one other is the Parkinson’s KinetiGraph. We compare our algorithm with the MDK algorithm by computing the percentage of each 1 min period that our algorithm detects tremor.

## 3. Results

### 3.1. Short-Time Fourier Transform vs. Continuous Wavelet Transform

**Figure 2:**
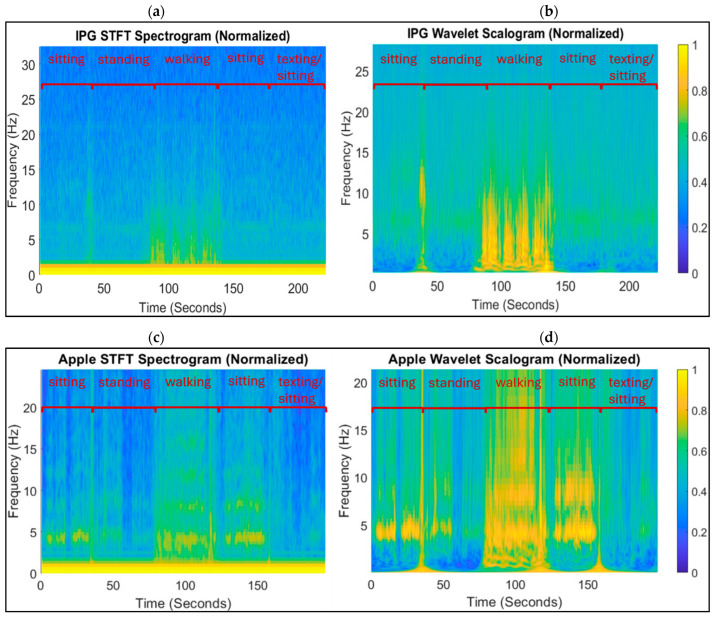
Spectral power density over time of acceleration signals obtained from the accelerometer implanted in the IPG (**a**,**b**) and that worn around the wrist in the Apple Watch (**c**,**d**). The spectral power estimates obtained by STFT (**a**,**c**) are compared with those obtained by CWT (**b**,**d**) and are shown for one exemplary clinical test session for Participant RZCH. Each scalogram and spectrogram is normalized to the maximum intensity for the given session to show relative peaks.

To obtain the spectral features on which our tremor algorithm is based, we first attempted STFT and integrated the spectrum obtained between the 4 and 7 Hz bands. A sample spectrogram and scalogram, from which peaks in the 4–7 Hz band are detected, are shown in Figure 2. Figure 2a and b was obtained from applying the STFT and CWT, respectively, to the IPG acceleration signals. Figure 2c,d was obtained from applying them to the Apple Watch acceleration signals. Ultimately, the CWT provided better frequency resolution and dynamic range so that large spikes in amplitude in a given frequency do not pose miscalibration for peak detection thresholds. Tremor peak detection could be obscured by periods of activity such as transitions between sitting and standing. However, the CWT, known for its better dynamic range, provided the spectral features with greater contrast of peaks in frequency and time-frequency resolution, as can be seen in Figure 2. The improved contrast in the CWT scalogram over the STFT spectrogram was noticeable whether using the IPG accelerometer (Figure 2a,b) or Apple Watch accelerometer (Figure 2c,d).

**Figure 3:**
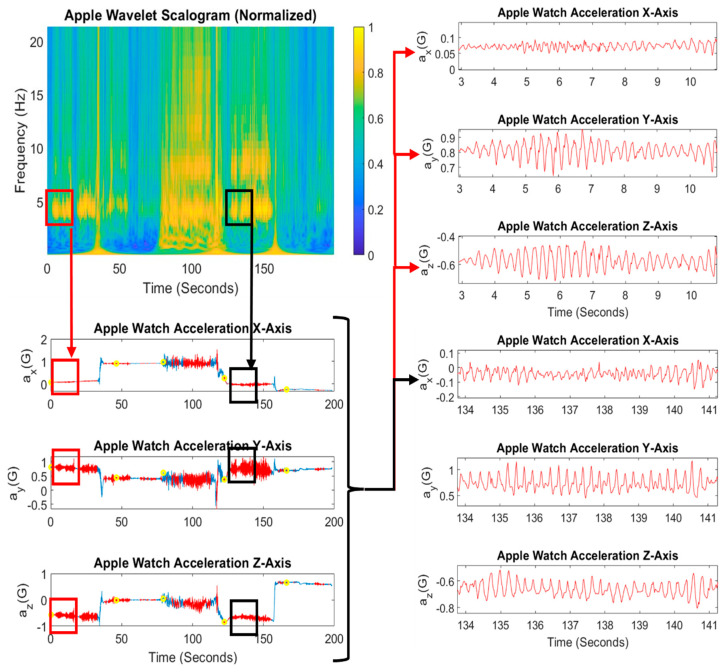
The raw Apple Watch acceleration signals aligned in time with corresponding CWT scalogram, illustrating that the CWT scalogram reveals distinct peaks in the 4–7 Hz band when there are clear Parkinsonian tremor oscillations in the acceleration signals. The scalogram is normalized to the maximum intensity for the given session to show relative peaks.

Figure 2 also illustrates the ability to detect the fundamental frequency more accurately with CWT when harmonics are present due to the better frequency resolution and dynamic range. This in turn allows for distinguishing between the walking state and tremor state, and provides the capability to detect the presence of tremor during walking. Figure 3 shows the same CWT scalogram of the Apple accelerometry signal with the acceleration signals aligned in the time domain. The insets show places where the presence of tremor was not obvious in the time domain signal, and also less obvious in the STFT (Figure 2c), but when zooming in on portions of the signal when clear 4–7 Hz peaks are visible in the CWT scalogram, Parkinsonian tremor is evident in the time domain.

### 3.2. Implanted vs. Wrist-Worn Accelerometry for Tremor Detection

The tremor algorithm was applied to both sets of acceleration signals—from the IPG and from the Apple Watch. Comparing Figure 2b,d, peaks in the 4–7 Hz band when tremor exists, such as during the sitting phases, were on average 60% more intense in amplitude in the CWT scalogram obtained from the Apple Watch than the IPG. Zooming in on the time domain acceleration signals, as shown in Figure 3 insets, reveals that indeed tremor does exist at the times that the tremor is detected using the Apple Watch scalogram. Also, comparing Figure 2b,d reveals that the tremor band harmonics show up more readily during walking, in addition to harmonics of the walking rhythm itself, which allows for more accurate detection of tremor during walking, whereas the IPG scalogram would only reveal walking but could not be disambiguated from tremor during walking. This could be explained by the difference in anatomical location of the two sensors: the IPG implanted in the trunk of the body (subclavicular) and the Apple accelerometer worn on the wrist. Parkinsonian tremor typically is observed in the distal limbs [29] but there were potential factors that could have disadvantaged the use of a wrist-worn accelerometer—namely, that it is more prone to motion artifacts and extrinsic noise—and the IPG accelerometer had a higher temporal resolution. Despite those factors, Parkinsonian tremor shows up more clearly in the Apple acceleration, and tremor harmonics are more readily discriminable from the Apple acceleration spectra. Thus, it appears that positioning the sensor on the appendages overrides the advantages of the implanted accelerometer for the purposes of detecting Parkinsonian tremor. We have observed that due to the IPG accelerometer’s location in the trunk of the body, it is better suited for classifying different physical activity states [30].

### 3.3. Comparison of CWT-Based Tremor Metric with MDK Tremor Metric

We compared our tremor metric to the MDK tremor metric. We first attempted to use the MDK metric for validation, but through careful visual inspection, we contend that we have developed a metric that has relatively high accuracy at a 1 s resolution. Visual inspection was carried out by a trained research scientist, who has over 8 years of experience specifically analyzing the motor signals and behavior of patients with DBS for Parkinson’s disease. He identified signal portions that were periodic in nature, counting cycles and dividing by the duration of the regular oscillations to verify that the oscillations occurred in the 4–7 Hz range, and counted them as tremor detection if the amplitude of the oscillations was above 0.1 g (gravities, the units of the Apple Watch accelerometer), or 1 m/s^2^, which is the clinical standard for classifying mild vs. moderate tremor. Here, we examine a couple of illustrative cases.

**Figure 4:**
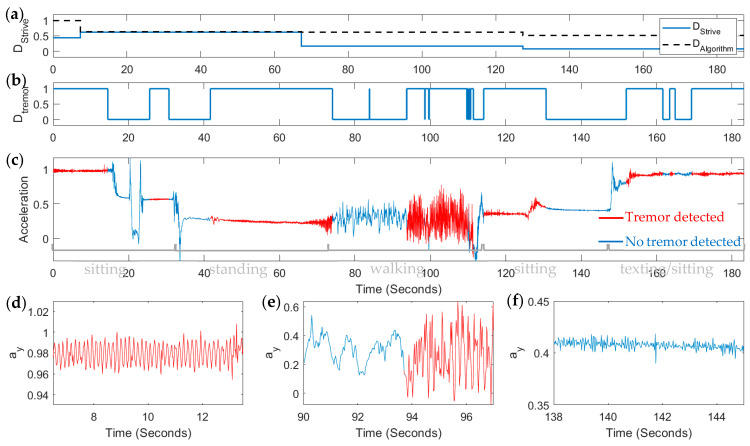
Sample output showing performance of our tremor detection algorithm. (**a**) The Apple movement disorders kit (MDK) tremor metric (solid trace) is provided as a ratio of time in a minute that tremor is found to be present. Our detected tremor metric Dtremor was integrated over 1 min periods and shown also as a ratio of time in a 1 min period during which tremor was found to be present. (**b**) Our detected tremor is computed on a second-scale resolution. (**c**) The corresponding acceleration signal is shown vs. time along with the physical activity state of the participant written in text below. The signal is color coded to show when tremor was detected by our algorithm (in red) and not detected (in blue). (**d**) Acceleration signal is shown, at a higher resolution time scale, during the period from about 7 to 13 s during which our algorithm detected tremor present; (**e**) during a period when the participant is walking and our algorithm correctly detects the transition from no tremor present to tremor onset; and (**f**) during a period when the participant is sitting and no tremor is present, as our algorithm again accurately detects.

Figure 4 shows an example of the detected tremor D_tremor_ detected by our detection algorithm, and how it compares with the raw acceleration signal in time. We are able to accurately detect periods of tremor (detections by our algorithm shown in red) as visually verified by zooming in on the time axis to observe periods when the regular oscillatory pattern of tremor is readily evident (Figure 4d), during transitions from periods without tremor to periods with tremor (Figure 4e) and periods where it is evident that there is no tremor (Figure 4f). Our detection algorithm accurately detects these occurrences of tremor on a second-time resolution, whereas the MDK tremor metric (labeled D_Strive_ in Figure 4a, solid trace) is updated every minute. We derived a comparable metric D_Algorithm_ from our own finer time-resolved metric by integrating our D_tremor_ signal over 1 min periods and dividing by the number of samples in one minute. This gave us the percentage (or technically the ratio) of time in a one-minute period during which tremor was detected. In the example shown in Figure 4a, our tremor metric tended to pick up more tremor than the MDK tremor metric. Most notably, our algorithm detects more of the tremor that is present during other activity (such as walking and texting) than the MDK tremor metric. It is able to disambiguate between walking and tremor and walking with tremor.

**Figure 5:**
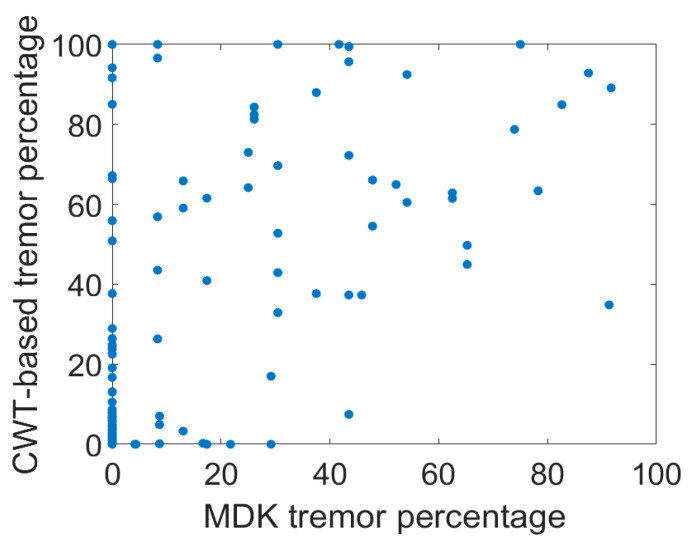
Scatterplot of percent tremor according to our CWT-based detection algorithm versus that of the MDK algorithm. There is a general positive correlation but clearly substantial discrepancies between the two algorithms’ estimations. The MDK algorithm has an indeterminate case which it labels as “unknown” and appears as “0%” tremor. Many of the discrepancies with our algorithm occur when MDK estimates the patient tremor state to be “unknown”.

The scatterplot of our D_Algorithm_ vs. MDK’s D_Strive_ tremor percentages is shown in Figure 5. The Pearson’s correlation coefficient was determined to be 0.57 (t-value = 8.5, dof = 148, *p* < 1 × 10^−6^).

As aforementioned and illustrated in Figure 4, upon close visual inspection, we found many cases of our spectral feature-based algorithm correctly detecting walking alone and tremor during walking, whereas MDK’s algorithm would often miss tremor during walking or incorrectly detect walking as tremor. There were other instances in which substantial tremor was missed by MDK’s algorithm but accurately detected by ours. Finally, for one participant in particular, AC27, our algorithm detected tremor during texting; however, upon closer inspection and comparing against logs that were recorded during the experimental sessions, our algorithm appeared to generate false positives for tremor when the participant exhibited dyskinesia.

While the MDK algorithm cannot be used as a clinical gold standard for the aforementioned reasons, the comparison and resulting mismatches with our CWT-based algorithm highlight our algorithm’s ability to detect tremor during walking and reveal a need for further study and development of tremor detection algorithms that can discriminate tremor from texting and dyskinetic movements. Table 2 is an atypical confusion matrix, in that it is simply comparing two detection algorithms, as no gold standard ground truth is available. Since the metric provided by MDK was a percentage of time over 1 min periods during which tremor was detected, we set a threshold of 20% to define whether tremor was detected or not detected. According to this definition, and using MDK as an industry standard reference, the “correct detection rate”, or match rate between the two algorithms, was 78.3%; the “sensitivity”, indicating how many of the MDK detections our algorithm also detected, was 90.5%; and the “specificity”, indicating how our algorithm’s identification of periods without tremor matched MDK’s, was 73.6%.

### 3.4. Tremor Detection Correlates with DBS Intensity (Increased Tremor Detection with Decreasing DBS Intensity)

**Figure 6:**
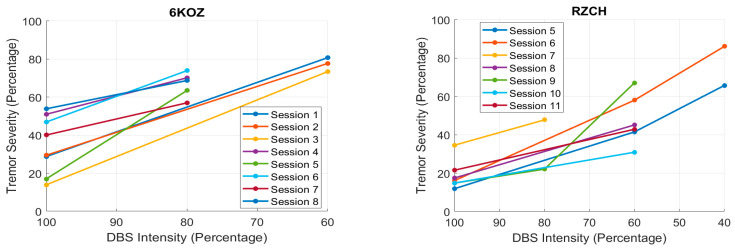
The tremor severity estimated by our algorithm, calculated as the percentage of time during a session that tremor is detected vs. DBS intensity, represented as a percentage of the prescribed DBS intensity (100%) which is meant to minimize Parkinson’s symptoms. Tremor severity was computed by summing our binary metric (D_tremor_) over a given session and dividing by the total number of samples. Each trace represents one session during which the stimulation intensity was changed to multiple levels. Shown for two participants whose dominant PD symptom is tremor. Tremor severity increases with decreasing stimulation intensity.

When permissible for a given participant at a given clinical research visit, the acceleration data were collected for the 2.5 min activity sequence at different DBS intensities, 100%, 80%, and 60%, with respect to their normal prescribed DBS settings (for optimal efficacy of symptom control vs. side effects). Thus, we had the potential to determine any relationship between DBS intensity and tremor severity, as detected by our tremor detection algorithm. We compared our metric to the StrivePD implementation of the Apple MDK tremor severity metric. Since the two metrics were on different time scales, we first computed a comparable metric by integrating over the same 1 min periods as the MDK samples. We computed the percentage of time during a minute in which tremor was detected by our algorithm for all the sessions available during a 12-month period for participants who were able to perform the activity sequence at multiple DBS settings within a given session (day). If the experimenter deemed that the participant should not have the DBS intensity reduced further, due to the observed symptom severity, the DBS setting was not modified further (and the DBS intensity remained at 100% or 80%). Only two participants had several sessions in which the activity sequence was repeated at multiple DBS settings. Scatterplots of their detected tremor severity vs. the DBS intensity for these two participants are shown in Figure 6. These scatterplots show that the tremor severity, according to our detection algorithm, increases with decreasing DBS intensity. The repeated measures correlation coefficients [31] of −0.93 (df = 9, *p* = 3.6 × 10^−5^) for RZCH, −0.97 (df = 7, *p* = 1.6 × 10^−5^) for 6KOZ, and r = −0.94, *p* = 0.057 for NU5U indicates a strong and statistically significant negative correlation between DBS intensity and tremor severity, as detected by our tremor detection algorithm.

## 4. Discussion

In the present work, we present an automated tremor detection algorithm that is able to detect tremor using a wrist-worn accelerometer even in the presence of other daily living activities, such as standing, walking, sitting, and texting. It is able to detect tremor on the order of seconds, besting the current industry standard, which makes detections on a minute scale, by an order of magnitude. The tremor severity detected by our algorithm is significantly correlated with DBS intensity, showing its clinical relevance. Our algorithm may be able to more accurately detect tremor during walking and walking without tremor. This is important for multiple reasons, including the common phenomenon for hand tremor to become severe while walking as the individual’s attention is diverted away from hand control to walking [32,33]. Also, this tremor detection algorithm would be integrated into a physical activity classifier that would provide more precise control tailored to the patient’s continuously changing needs, whether it is tremor suppression, gait support, balance, or postural support.

While our detection algorithm is distinct from the MDK algorithm in its ability to discriminate walking from tremor, we discovered through comparison with the test administrator’s logs that there are two other types of motor activity that generate false positives for tremor according to our algorithm. (1) Upon close visual inspection, there was oscillatory activity in the 4–7 Hz frequency range for two subjects during texting. An investigation in the literature revealed that a 5 Hz brain rhythm has been exhibited during texting [34,35]. (2) For AC27, as aforementioned, our algorithm detected false positives when dyskinesia was present. The “signature” for dyskinesia and any distinguishing trademarks from those for tremor will be the subject of further study.

The automated and continuous estimates of tremor could support progress towards the development of adaptive DBS, so that DBS will more efficiently and effectively manage and target symptoms depending on the individual’s moment by moment needs. In our prior work [30], we have begun developing an activity classification algorithm that performs with greater accuracy when utilizing the implanted (IPG) accelerometer. However, the presence of tremor confounds walking and standing cluster-based classification. Therefore, our overall physical activity classifier will employ a separate tremor detection algorithm to first test to see if tremor is present. Tremor will be one of the activity state classifications; if tremor is not present, then the algorithm will proceed with clustering to determine if the current state is resting, standing, or walking.

Further testing is needed to determine whether our algorithm is generalizable and to fully develop our automated tremor detection algorithm. The present study was limited by a small cohort within a restricted age range of 60–70 years, with a specific medical indication for DBS implanted in both the subthalamic nucleus (STN) and globus pallidus interna (GP) with distinctly low dosing for PD medication (namely, levodopa) [13]. To determine the generalizability of our algorithm, it needs to be validated in subjects across a wider age range and DBS electrode location and more subjects of different symptom profiles, as the manifestation of symptoms and how a patient’s symptoms respond to DBS will vary from person to person. The outcomes can be influenced by individual differences, including age, and the stimulation targets [36,37,38]. We envision this tremor detection algorithm to be eventually implemented in an adaptive DBS controller that would adapt stimulation based not only on tremor but on a spectrum of PD symptoms. As DBS target location is a key factor of variability across individuals and could certainly affect even the manifestation of tremor in measured acceleration, we would need to test the algorithm in PD patients across a wide spectrum of symptom profiles and DBS targets. As aforementioned, this present study was part of a larger feasibility study [13] specifically investigating DBS at a combination of STN and GP targets; hence, our subject pool was restricted to STN and DBS targets. Out of the three participants with dominant tremor symptoms at home, only two had sufficient data; there were technical difficulties with the third participants’ Apple Watch data stream, which meant that we only had five data points in the 1-year period for this participant and did not have quite sufficient data to reach statistical significance, although this participant’s tremor percentage also was strongly correlated with DBS level (r = −0.94). As with all tremor detection studies, our validation results are limited by the absence of an objective quantitative ground truth metric. In our future work, we plan to compare our expert visual inspection of acceleration signals, as described in the Methods, against a clinician’s continuous visual observation during daily living activities, akin to a UPDRS tremor scale rating but continuously over a longer stretch of time at a much higher temporal precision and during daily living activities. Our original quest was to determine if we could accurately detect tremor from the IPG accelerometer, but our investigation indicates that the most accurate tremor detection will require an external wrist-worn accelerometer which poses an additional challenge to developing a fully embedded aDBS controller. Despite these limitations on the ability to generalize our results, we believe that our algorithm represents a useful tool for further studies of the interaction between tremor, naturalistic movement and DBS.

In prior work [30], we developed a clustering classification algorithm based on spectral features which could classify three different physical activity states: (1) walking, (2) standing, or (3) sitting/resting. However, the results were confounded when tremor was present. Therefore, in our present work, we developed a tremor detection algorithm designed to be applied first, so that only if tremor is not present would we apply our physical activity state classifier and stimulation could be adjusted to the severity of tremor. Our future work will entail incorporating the Apple Watch-based tremor detection into our physical state classifier and testing the overall classifier on the data obtained in the clinic. The ability to detect tremor, and eventually other physical states, on a second scale will allow the DBS controller to provide tremor suppression, postural support, gait support, and the like, according to the patient’s moment-by-moment changing needs. The second-scale time resolution is also expected to better equip our algorithm with the ability to detect re-emergent tremor in PD, which typically occurs after a posture is held for a few to several seconds [39]. We also plan to implement our tremor detection in embedded hardware and test the automated calibration algorithm.

## 5. Conclusions

In conclusion, we have developed a tremor detection algorithm that utilizes continuous wavelet transform-based spectral analysis on triaxial acceleration from a commercially available Apple Watch. Importantly, although the results of our low-sample study are limited, the algorithm is shown to be able to distinguish between voluntary physical activity including walking and tremor and make estimates on a second scale. The continuation of our work will include integrating our tremor detection algorithm in a physical activity classifier that will advance the prospects for closed-loop adaptive DBS for Parkinson’s disease.

## Figures and Tables

**Table 1:** Participant demographic and baseline data. Note: Years since diagnosis reported at the start of the data collection.

Participant ID	Age	Sex	Yrs Since Diagnosis	OFF UPDRS Tremor Score ^1^	ON UPDRS Tremor Score
AC27	66	F	22	0	0
E395	65	M	15	3	0
NU5U	60	F	14	5	4
RZCH	69	M	12	7	6
6KOZ	70	M	14	5	4

^1^ UPDRS-III scores ON/OFF medications without DBS. Values are summed over all subitems 20 and 21 of the UPDRS-III.

**Table 2:** Confusion matrix comparing our CWT-based algorithm against MDK’s algorithm. Detection of tremor was defined as the percentage of time over a 1 min period during which tremor was detected exceeding 20%.

		our CWT-based algorithm	
		Detected	Not Detected
MDK algorithm	Detected	38	4
	Not Detected	29	81

## Data Availability

To preserve the anonymity of the participants, participant data are available upon request and a data use agreement. All data necessary to evaluate the claims within this work are included.

## Abbreviations

The following abbreviations are used in this manuscript:

CWT	Continuous wavelet transform
STFT	Short-time Fourier transform
MDK	Movement disorders kit
DBS	Deep brain stimulation
